# 1029. Increased Mortality in Hospital-Onset Carbapenem-Resistant Enterobacterales Bloodstream Infections

**DOI:** 10.1093/ofid/ofad500.060

**Published:** 2023-11-27

**Authors:** Angelique E Boutzoukas, Lauren Komarow, Natalie A Mackow, Abhigya Giri, Liang Chen, Carol Hill, Yohei Doi, Michael J Satlin, Cesar A Arias, Minggui Wang, Laura Mora Moreo, Erica Herc, Eric Cober, Gregory D Weston, Vance G Fowler, David van Duin

**Affiliations:** Duke University, Durham, North Carolina; George Washington University, Rockville, Maryland; University of North Carolina, Chapel Hill, NC; The George Washington University, Rockville, Maryland; HMH-CDI, Nutley, New Jersey; Duke Clinical Research Institute, Durham, North Carolina; University of Pittsburgh, Pittsburgh, PA; Weill Cornell Medicine, New York, NY; Houston Methodist and Weill Cornell Medical College, Houston, TX; Institute of Antibiotics, Huashan Hospital, Fudan University, Shanghai, Shanghai, China (People's Republic); Department of Health Services Research and Policy, Faculty of Public Health and Policy, London School of Hygiene & Tropical Medicine, London, England, United Kingdom; Henry Ford Hospital, Detroit, Michigan; Cleveland Clinic Foundation, Cleveland, OH; Montefiore Medical Center and Albert Einstein College of Medicine, Bronx, NY; Duke University Medical Center, Durham, NC; University of North Carolina at Chapel Hill, Chapel Hill, NC

## Abstract

**Background:**

Hospital-onset (HO) carbapenem-resistant Enterobacterales (CRE) infections are increasing; recently, the Centers for Disease Control and Prevention (CDC) reported a 35% increase during the COVID-19 pandemic. We evaluated the impact of HO CRE blood stream infections (BSI) on outcomes compared to community-onset (CO) CRE BSI.

**Methods:**

Patients prospectively enrolled in CRACKLE-2 from 56 hospitals in 10 countries between April 30, 2016 to November 30, 2019 with a qualifying CRE bloodstream culture were eligible. Infections were defined per CDC guidelines as CO when the culture was obtained immediately prior to admission or through the first three days of hospitalization, and HO when the culture was obtained on or after the fourth day of admission. Categorical variables were tested using a chi-square test; continuous variable distributions were compared using Wilcoxon rank sum and Kruskal Wallis tests, as appropriate. The primary outcome was desirability of outcome ranking (DOOR) 30 days after index culture. Difference in 30-day mortality was calculated with 95% confidence intervals (CI).

**Results:**

Among 891 patients with CRE BSI (Table 1), 65% of BSIs were hospital-onset (582/891). Compared to those with CO CRE, patients with HO CRE were younger (median 60 [Q1 42, Q3 70] years vs 65 [52, 74]; p< 0.001), had lower Charlson comorbidity score (median 2 [1, 4] versus 3 [1, 5]; p=0.002), and more often had Pitt bacteremia sore ≥4, indicative of critical illness, (47% versus 32%; p=< 0.001). ICU admission prior to first culture was associated with HO BSI (68% versus 42%, p< 0.001). Distribution of DOOR outcome at 30 days is shown in **Figure 1**. The probability of a better DOOR outcome in a randomly selected patient with CO BSI compared to a patient with HO BSI is 60.6% (95% CI: 56.8-64.3%). Mortality at 30-days was 11.6% higher in HO BSI than CO BSI (95% CI: 5.7, 17.6%, p=0.0003). A Kaplan-Meier curve of all-cause 30-day mortality is in **Figure 2**

**Figure:**
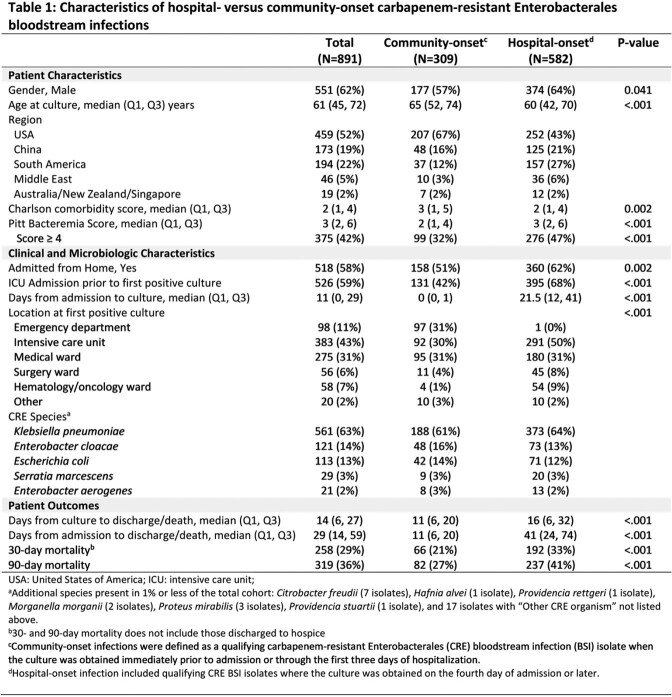

**Figure 1:**
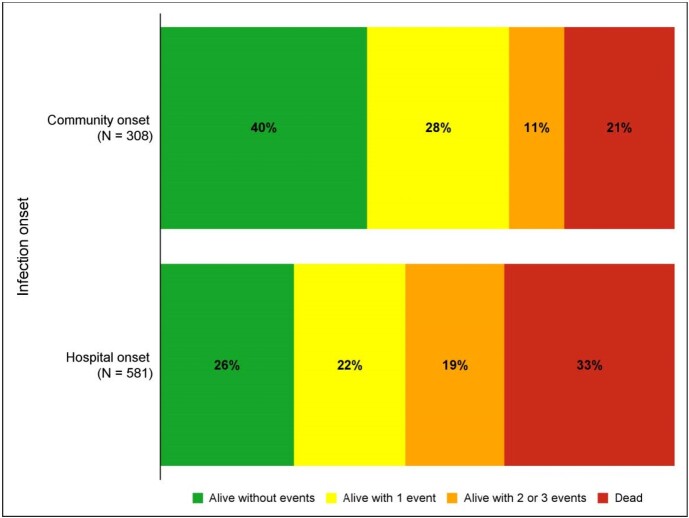
Distribution of Desirability of Outcome Ranking (DOOR) Categories by Infection Onset

Desirability of outcome ranking at 30-days. Events evaluated included 1) deleterious effects, including absence of clinical response, prolonged hospitalization (>30 days following first positive culture), or readmission within 30 days; 2) any adverse events including renal failure or C. difficile infection; and 3) mortality at 30 days. Two subjects (1 in hospital onset group and 1 in community onset group) missing 30-day DOOR due to missing disposition information are not included in the figure.

**Figure 2:**
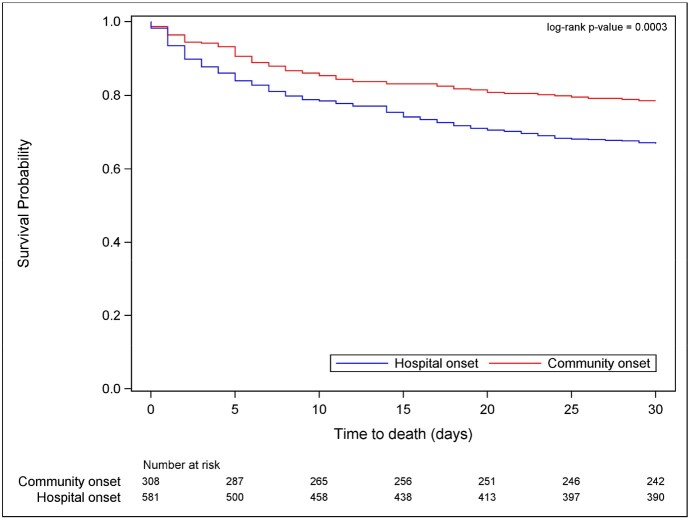
30-day Survival Plot by Infection Onset

Two subjects (1 in hospital onset group and 1 in community onset group) are excluded from the figure due to missing mortality outcome information. One subject in the hospital onset group died on day 30 and is still considered at risk in the figure.

**Conclusion:**

HO CRE BSIs are associated with worse outcomes than CO CRE BSIs; this unique population warrants special attention. As CRE often contain mobile genetic elements that facilitate horizontal transfer, close monitoring of HO CRE rates and optimizing hospital infection prevention methods are critical to prevent added morbidity and mortality.

**Disclosures:**

**Yohei Doi, MD, PHD**, bioMerieux: Advisor/Consultant|FujiFilm: Advisor/Consultant|Gilead: Advisor/Consultant|Gilead: Honoraria|GSK: Advisor/Consultant|Meiji Seika Pharma: Advisor/Consultant|Moderna: Advisor/Consultant|Moderna: Honoraria|MSD: Advisor/Consultant|MSD: Honoraria|Shionogi: Advisor/Consultant|Shionogi: Grant/Research Support|Shionogi: Honoraria **Michael J. Satlin, MD**, AbbVie: IDMC member|Biomerieux: Grant/Research Support|Merck: Grant/Research Support|SNIPRBiome: Grant/Research Support **Vance G. Fowler, MD, MHS**, Amphliphi Biosciences, Integrated Biotherapeutics; C3J, Armata, Valanbio; Akagera, Aridis, Roche, Astra Zeneca: Advisor/Consultant|Genentech, Regeneron, Deep Blue, Basilea, Janssen;: Grant/Research Support|Infectious Diseases Society of America: Honoraria|MedImmune, Allergan, Pfizer, Advanced Liquid Logics, Theravance, Novartis, Merck; Medical Biosurfaces; Locus; Affinergy; Contrafect; Karius;: Grant/Research Support|Novartis, Debiopharm, Genentech, Achaogen, Affinium, Medicines Co., MedImmune, Bayer, Basilea, Affinergy, Janssen, Contrafect, Regeneron, Destiny,: Advisor/Consultant|Sepsis diagnostic: Patent pending|UpToDate: Royalties|Valanbio and ArcBio: Stock Options **David van Duin, MD, PhD**, Entasis: Advisor/Consultant|Merck: Advisor/Consultant|Merck: Grant/Research Support|Pfizer: Advisor/Consultant|Pfizer: Honoraria|Qpex: Advisor/Consultant|Roche: Advisor/Consultant|Shionogi: Advisor/Consultant|Shionogi: Grant/Research Support|Union: Advisor/Consultant|Utility: Advisor/Consultant

